# Prevalence of HCV Infection in Household Contacts of Chronic Liver Diseases Cases in Egypt

**DOI:** 10.1155/2018/2153537

**Published:** 2018-10-24

**Authors:** Hanaa E. Bayomy Helal, Abdelmoniem Yuonis, Rania H. M. Shaker, Mona Ahmed Elawady

**Affiliations:** Department of Public Health and Community Medicine, Banha Faculty of Medicine, Banha, Egypt

## Abstract

**Background:**

Egypt has the highest prevalence of HCV infection worldwide. This project aimed at identifying the role of HCV transmission among household contacts to index cases in the persistent high incidence of HCV infection in Egypt.

**Methods:**

This cross-sectional study recruited 70 Egyptian cases with chronic liver diseases and their household contacts (140 contacts) from Qalubeyia Governorate. An interview questionnaire was used to collect information on sociodemographic characteristics and risk factors to HCV infection. HCV-RNA was tested using real-time polymerase chain reaction (PCR). Univariate and multivariate analyses were carried out to estimate the risk of HCV infection among contacts.

**Results:**

HCV viremia was detected in 85.7% of cases and 20% of contacts. HCV-RNA was detected in higher proportion of household contacts to cases than the general population. Contacts to HCV-positive cases were unlikely exposed to used syringe (*P*=0.02) and unlikely to have history of Bilharziasis (*P*=0.001) compared to contacts to HCV-negative cases. HCV-positive contacts were more likely older (*P* < 0.001) and married (*P*=0.008) and had higher crowding index (*P*=0.04) than HCV-negative contacts. Also, HCV-positive contacts were more likely exposed to blood transfusion (*P*=0.008) and shaving at community barber (*P*=0.04) and had history of Bilharziasis (*P*=0.01). The strongest predictors for HCV infection among contacts were old age (OR, 95% CI: 1.08, 1.02 to 1.15; *P*=0.01) and blood transfusion (8.08, 1.75 to 37.3; *P*=0.007).

**Conclusion:**

Nonetheless, household contacts to HCV cases are exposed to increased risk of HCV infection, and environmental exposure particularly blood transfusion remained a major source of HCV infection.

## 1. Introduction

In 2008, the Egyptian Demographic Health Survey reported that the prevalence of hepatitis C virus (HCV) infection was 14.7%, which was the highest prevalence of HCV in the world. [1] In 2013, the prevalence of HCV viremia was 7.3% due to the high mortality among old people with high prevalence of infection. [2] While in 2015, the prevalence of active HCV-infected cases was 4.4 [3].

A new era in the elimination of viral hepatitis has launched in 2015 when the United Nations General Assembly adopted the 2030 Agenda for Sustainable Development, which called on the international community to combat hepatitis. Viral hepatitis is a major public health challenge worldwide. The World Health Organization Eastern Mediterranean Region and the European Region have the highest reported prevalence of HCV particularly in the low- and middle-income countries. Globally, in 2015, the disease caused 1.34 million deaths. Each year, 1.75 million people newly acquire hepatitis C virus infection. These people are at risk of a slow progression to severe liver disease such as cirrhosis, hepatic decompensation, and hepatocellular carcinoma and death, unless they receive timely testing and treatment. [4].

Unsafe blood contact is the main source of HCV infection [5]. In Egypt, the main routes of HCV transmission have included the mass parenteral antischistosomal treatment from the 1950s to the 1980s, shared and reused needles, inadequate sterilization during dialysis, surgery, and dental care, and unsafe blood transfusion. Unsafe healthcare-related injections have been the major route of HCV transmission [6, 7]. Although the provision of safe injection practices was associated with reduced occurrence of HCV infection [8], the global incidence rate remained high (23.7 per 100,000) [4].

In Egypt, the prevalence of HCV infection is very high, and direct healthcare costs for hepatitis already consume 4% of the total health expenditure. Moreover, the indirect costs represent twice the direct costs. HCV treatment is highly cost-effective. That treating 328,000 HCV-infected patients annually by 2018 with the direct-acting antivirals (DAAs) could reduce the prevalence of infection by 94% and liver-related deaths by 75% by 2030 [9].

In Egypt, hepatitis C is just a problem in every family. The high prevalence of HCV infection and the clustering effect observed between HCV infections in households were linked to parenteral treatment for schistosomiasis [10]. However, the high incidence rate remained a pressing challenging to the already overstretched Egyptian healthcare system. Intrafamilial transmission has been accused for this high incidence. There are two patterns of intrafamilial transmission: horizontal transmission between patients infected with HCV and their household contacts sharing the same residential space due to shared behaviours and living conditions, and vertical transmission through the perinatal route. The perinatal transmission was found to have a minimal role [11, 12].

This project aimed to identify the role of intrafamilial transmission (transmission in relatives living in the same household) in the persistent high incidence of HCV infection in Egypt.

## 2. Subject and Methods

This cross-sectional study was conducted on cases with chronic liver diseases who were referred to the Molecular Biology Unit at Benha Faculty of Medicine, Qalubeyia Governorate, Egypt, to test for HCV infection and their household contacts. The field work was carried out over the period between September 2017 and February 2018.

An approval from the Research Ethics Committee in Benha Faculty of Medicine was obtained to conduct this work. A written informed consent (in Arabic language) was obtained from all participants. It included all details about the study (title, objectives, methods, expected benefits and risks, and confidentiality of data).

### 1.1. Subjects

The least number of contacts was determined according to the following equation:

(1) $$\begin{array}{c} \text{sample}\;\;\mathrm{size}=\frac{{\left({\text{Z}}_{1-\propto /2}\right)}^{2}P\left(1-P\right)}{d^{2}}, \end{array}$$

where Z_1−∝/2_ is the standard normal variate at 5% type 1 error (*P* < 0.05); it is 1.96. *P* is the expected proportion based on previous studies (the prevalence of HCV viremia was 4.4% [3]). *d* is the absolute error (0.05).

According to the above equation, the minimal sample size was calculated as 123 after allowance for double the prevalence of HCV viremia in household contacts compared to the general population [1]. The study recruited 140 contacts to 70 HCV-infected cases over the period of the study.

### 1.2. Inclusion Criteria

Cases with chronic liver diseases and aged ≥18 years old (to increase the likelihood of HCV infection) and their household contacts of both sexes and any age who were living in the same household with a case for at least one year were the candidates for the study.

### 1.3. Data Collection Tools

An interview questionnaire, which was reviewed and approved in previous studies [13–15], was used to collect information on personal data, socioeconomic characteristics, present and past health, and risk factors for exposure to HCV infection including history of surgical operations, dental procedures, blood transfusion, schistosomiasis treatment, contaminated needles or puncture, prior hospitalization, shared use of toothbrushes or shaving razors, common tools for nail trimming, circumcision, condom use, drug abuse, smoking, wet cupping (higama), tattooing, and multiple sexual partners.

At the same visit, venous blood samples were collected to test for HCV-RNA using the quantitative real-time polymerase chain reaction (PCR). Extraction of viral RNA by the QIAamp Viral RNA mini kit was carried out using the QIAcube automatic extractor (QIAGEN GmbH). Amplification by TaqMan PCR master mix artus HCV RG RT-PCR kit (QIAGEN GmbH) was performed using the real-time PCR machine (Applied Biosystems StepOne Real-Time PCR System, San Diego, Ca, USA).

A pilot study was undertaken on 10 subjects (including equal numbers of males and females), and their questionnaires were not included in the study. Testing of the questionnaire was useful in estimating the time taken to answer the questions and understanding of the questions. This helped to reduce limitations of understanding as well as nonresponse to questions.

### 1.4. Statistical Analysis

Mean ± standard deviation (SD) and range were used to describe quantitative data, and frequency and percentage were used to describe qualitative data. Comparisons between the different study groups were carried out using the chi-square test and the Fisher exact test to compare proportions as appropriate. The Student *t*-test was used to measure the mean difference between two groups regarding parametric data. Multiple logistic regression analysis for HCV-positive contacts conditioned on being a contact of an HCV case and other potential risk factors was carried out. The risk of HCV infection was presented as odds ratio (OR) and 95% confidence interval (CI). Statistical significance was accepted at *P* < 0.05. All statistical analyses were conducted using STATA/SE version 11.2 for Windows (STATA corporation, College Station, Texas).

## 3. Results

Study participants comprised 70 cases with chronic liver diseases, their ages ranged between 19 and 78 years with a mean of 49.5 ± 11.7 years, and males constituted 60% of them. HCV-RNA was detected in 60 cases (85.7%). Household contacts comprised 140 cases, their ages ranged between 3 and 75 years with a mean of 33.28 ± 16.75 years, and males constituted 40.71 % of them. HCV-RNA was detected in 28 households (20%). Detailed description of the characteristics of studied cases and their households and exposure to risk factors for HCV infection are shown in Table 1.

**Figure 1:**
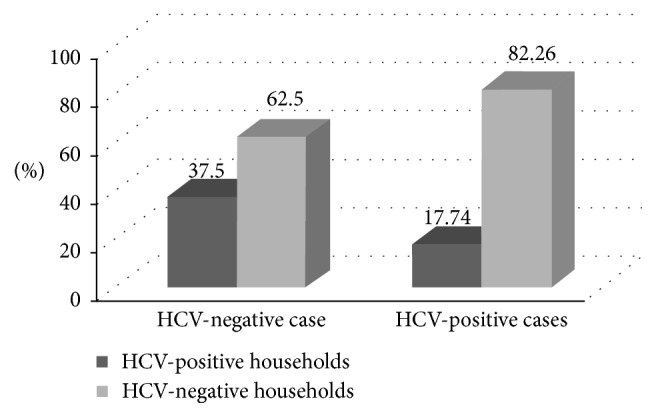
Relationship between studied cases and household contacts regarding the HCV infection.

Figure 1 shows the results of real-time PCR, and 6 out of 16 household contacts to HCV-negative cases (37.5%) had HCV viremia. While 22 out of 124 household contacts to HCV-positive cases (17.74%) had HCV viremia. However, this difference was nonsignificant (OR, 95% CI: 0.36, 0.1 to 1.35, and *P*=0.09).

Table 2 shows comparisons between household contacts to cases with HCV infection and those contacts to cases without HCV infection. Household contacts to HCV-positive cases were less likely to be exposed to used syringe than contacts to HCV-negative cases (0.12, 0.003 to 0.82, and *P*=0.02). Household contacts with history of Bilharziasis were less likely among contacts to HCV-positive cases compared to contacts to HCV-negative cases (0.08, 0.02 to 0.39, and *P*=0.001).

Comparisons between HCV-positive and HCV-negative household contacts were carried out as shown in Table 3. HCV-positive contacts were more likely older (*P* < 0.001) and married (*P*=0.008) and had higher crowding index (*P*=0.04). HCV-positive contacts were more likely exposed to blood transfusion (*P*=0.005) and shaving at community barber (*P*=0.04) and had history of Bilharziasis (*P*=0.01).

Multiple logistic regression analysis for being HCV-positive household contacts conditioned on exposure to a HCV-positive case and other potential risk factors was carried out (Table 4). Contacts to HCV-positive cases were less likely to be HCV infection (OR, 95% CI: 0.14, 0.02 to 0.75, and *P*=0.02). However, the risk of being HCV-positive was increased with the increase in age (1.08, 1.02 to 1.15, and *P*=0.01) and among contacts who had blood transfusion (8.08, 1.75 to 37.3, and *P*=0.007).

## 4. Discussion

### 4.1. HCV Infection Remained the Main Cause of Chronic Liver Diseases

This cross-sectional study was conducted to determine the prevalence of hepatitis C infection among household contacts to infected cases. The study examined 70 patients with chronic liver disease, of whom 60 were infected with chronic hepatitis C and 10 had liver disease due to causes other than HCV infection. In addition, 140 household contacts of the chronic liver diseases cases were included, of these 124 were contacts to the HCV cases (2.07 contacts per case), and 16 were contacts to the HCV‐negative cases (1.6 contacts per case).

The results of the study revealed that HCV infection remained the main cause of chronic liver diseases with a prevalence rate of 85.7% among cases, while the overall prevalence of HCV in the household contacts of both cases and controls was 20%.

Of the household contacts of HCV-infected patients, 22 out of the 124 contacts were HCV viremic (17.7%), compared to 6 out of the 16 contacts of HCV-negative cases (37.5%). The prevalence in both groups of contacts is much higher than the prevalence in the general population, which in similar age groups is in the range of 4%-5% viremic prevalence [3, 16]. This might suggest an increased risk of HCV infection among contacts to HCV-infected cases who serve as reservoirs of infection to their household contacts. This was also suggested by previous studies in Egypt [17, 18]. A prospective cohort of 6,734 anti-HCV-negative rural Egyptians detected 33 seroconversions. The strongest predictor for seroconversions was having anti-HCV-positive family member [17]. A comparative study of Egyptian families which included 90 families with index HCV-positive case (257 contacts) and 38 families with no index case (75 contacts) reported that 32 out of the 90 families with index cases had one or more HCV-positive contacts (38/257; 14.8%), while only two families with no index cases had HCV-positive contacts (3/75; 4%). Hence, intrafamilial transmission was thought to be a major underlying factor to the high prevalence of HCV infection in Egypt [18].

Regarding viremic contacts of HCV-negative cases, the six contacts had past history of hospitalization, surgical procedures, and exposure to used syringe and were circumcised by nonmedical personnel, and three of them had past history of injection treatment for Bilharziasis (unpresented data). These increased the proportion of viremic contacts of HCV-negative cases.

### 4.2. HCV-Positive Household Contacts Were More Likely in Sexual Partners and Siblings

In the present study, HCV-positive household contacts were more likely in sexual partners and siblings. HCV positivity was more frequent in older, married contacts with higher crowding index. This corresponds to findings reported by concurrent studies in Egypt, which linked the familial transmission of HCV with advanced age (≥40) and sexual partners [18, 19]. Correspondingly, a cross-sectional study of 175 Italian HCV-positive patients and their family members found that HCV-positive family members accounted for 8.9% (23/259) with the highest prevalence in sexual partners (12.1%). The prevalence of anti-HCV was more likely in older family contacts [20]. Again in Italy over the period from 1975 to 2003, a total number of 2856 of HCV-infected index cases were invited with their family members (number =13,440) to take part in a study to investigate risk factors for HCV transmission. The overall prevalence of HCV infection in family members was 2.1%, with the highest prevalence in sexual partners (13.8%) followed by offspring (2.3%) and parents and siblings (2.1%) [21].

However, there were no significant differences in the prevalence of HCV infection between contacts to HCV-positive and HCV-negative cases. This contradicts the above suggestion. But, the high prevalence of HCV infection among contacts to HCV-negative patients could be explained by the past history of hospitalization, surgical procedures, exposure to used syringe, circumcision by nonmedical personnel, and prior injection treatment for Bilharziasis.

Moreover, it was found that HCV-positive contacts were more likely exposed to sources of infections such as shaving at common barber, infected blood, and Bilharziasis. Thus, the risk of HCV infection increased with environmental exposure to potential sources of infection. Correspondingly, the risk of HCV infection was increased among sexual partners of the Italian index cases who were intravenous drug users (23.6%) compared to cases who acquired infection through transfusions (7.8%), which suggests a parenteral route of transmission [21].

In addition, a controlled historical cohort study was conducted in Iran to investigate the intrafamilial transmission of HCV infection among sexual and nonsexual contacts. Only 2.9% (7/270 subjects) of contacts to HCV cases and 1.1% (3/270 subjects) of contacts to noninfected controls had anti-HCV antibodies. Of these, two subjects among the unexposed group proved to be HCV-infected during the recombinant immunoblot assay (RIBA) and PCR, and both of them were IV drug users. Thus, there was no intrafamilial risk for HCV transmission, and sexual contact was more likely to stimulate the immune system rather than increasing the risk of HCV infection [22].

Moreover, in the present study, we found that risky behaviours such as using nonsterile syringes and exposure to Bilharzial infection were less frequent among household contacts to HCV-infected case. This might reflect their awareness about the ways of HCV transmission.

In this study, HCV infection in household contacts to index cases was associated with past history of blood transfusion. However, in this study, whether this transfusion was before or after blood donor screening policy was not verified, but still blood transfusion represents a main risk for HCV infection, which emphasizes more efforts for safe blood transfusion. Similarly, 72% of subjects with history of blood transfusion (54/75 subjects) were HCV positive in the cross-sectional survey of one thousand healthy blood donors volunteered in Kaser Al Ani hospital blood bank, Cairo, Egypt [23]. Correspondingly, transfusion was commonly reported as the main rout of HCV transmission among blood donors in Iran [24, 25] and in USA [26], in asymptomatic urban population of the State of Mexico with at least one risk factor [27], and in HCV-infected patients who were identified between 2001 and 2008 in the Northern California, USA [28].

However, in Egypt, the main risk factor for HCV infection was the traditional injection treatment of schistosomiasis [29–31].

### 4.3. Limitations

The main limitations of this study were the unknown duration of the disease for index case and recall bias due to the cross-sectional nature of the study, which also makes it inaccessible to assess the time relationship between exposure to risk factors including the index case and outcome (HCV seropositivity) unlike the prospective cohort design. Further large-scale prospective studies to investigate the viral sequencing between cases and contacts (seroconversion) are recommended to prove or disprove the contact transmission.

As far as information bias is concerned, this type of error could be avoided by using a well-established means of collecting data on past exposures and risk factors. Regarding selection bias, we collected information regarding the contacts of the index patients attending Benha Faculty of Medicine, and we believe that our population is a random sample of patients with HCV.

Another limitation of the study refers to the lack of assessment for HCV genotypes, so we were unable to address whether family members share the same genotype, and differing genotypes have differing rates of transmission.

## 5. Conclusion

The controversy on the intrafamilial transmission of HCV infection can be due to the different methods used to detect anti-HCV antibodies (first and second generations ELISA have lower sensitivity than the third-generation assays), different geographic areas, viremia levels, and sexual behaviours of the target population.

Finally, it can be concluded that household contacts to HCV cases are exposed to increased risk of HCV infection. This is favoured by environmental exposures to other sources of infections. Nonetheless, contacts to HCV patients are more likely aware of the different ways of HCV transmission and avoid exposure to sources of infection. In addition, prolonged exposure to infected cases can stimulate the immune system.

## Figures and Tables

**Table 1:** Characteristics of the studied groups.

Variable		Cases (number = 70)		Household contacts (number = 140)		Variable		Cases (number = 70)		Household contacts (number = 140)	
		Number	%	Number	%			Number	%	Number	%
Sex	Females	28	40.0	83	59.29	History of blood transfusion	No	59	84.29	127	90.71
	Males	42	60.0	57	40.71		Yes	11	15.71	13	9.29
Age (years)	Mean ± SD (range)	49.51 ± 11.71 (19–78)		33.28 ± 16.75 (3–75)		Priossr surgical procedures	No	2	2.86	16	11.43
Occupation	Housewife	8	11.43	39	27.86		Yes	68	97.14	124	88.57
	Specialist	44	62.86	45	32.14	Prior hospitalization	No	40	57.14	98	70.0
	Medical/paramedical	6	8.57	5	3.57		Yes	30	42.86	42	30.0
	Student	2	2.86	43	30.71	Circumcision	By medical personnel	6	8.57	27	19.29
	Worker	10	14.29	8	5.71		By nonmedical personnel	63	90.0	89	63.57
Educational level	Illiterate	1	1.43	7	5.0		No	1	1.43	24	17.14
	Read and write	31	44.29	89	63.57	Condom use	No	68	97.14	138	98.57
	Basic education	8	11.43	20	14.29		Yes	2	2.86	2	1.43
	Secondary school	5	7.14	11	7.86	Shaving at community barber (males only)	No	7	10.00	59	42.14
	High education	25	35.71	13	9.29		Yes	63	90.00	81	57.86
Marital status	Single	4	5.71	50	35.71	Nail trimming	Private tool	22	31.43	27	19.28
	Married	65	92.86	90	64.29		Common tools	48	68.57	113	80.71
	Widow	1	1.43	0	0.0	Smoking Shisha	No	60	85.71	134	95.71
Residence	Urban	60	85.71	118	84.29		Yes	10	14.29	6	4.29
	Rural	10	14.29	22	15.71	Sharing razors	No	24	34.29	60	42.86
Relation to cases	Partners	—	—	37	26.43		Yes	46	65.71	80	57.14
	Parents	—	—	9	6.43	Exposed to used syringe^*∗∗*^	No	24	34.29	46	32.86
	Sons	—	—	72	51.43		Yes	46	65.71	94	67.14
	Siblings	—	—	13	9.29	Exposed to used toothbrush	No	60	85.71	122	87.14
	Others	—	—	9	6.43		Yes	10	14.29	18	12.86
Crowding index (persons/room)	Mean ± SD (range)	1.16 ± 0.66 (0.3–3)				Practice of tattooing	No	56	80.0	126	90.0
HCV-RNA	Negative	10	14.29	112	80.0		Yes	14	20.0	14	10.0
	Positive	23	32.86	12	8.57	Exposed to blood	No	54	77.14	114	81.43
	UD	37	52.86	16	11.43		Yes	16	22.86	26	18.57
Symptoms^*∗*^	Diarrhea	15	21.43	—	—	History of Bilharziasis	No	54	77.14	128	91.43
	Abdominal pain	35	50.0	—	—		Yes	16	22.86	12	8.57
	Easy fatigue	30	42.86	—	—		No	54	77.14	132	94.29
	Yellow sclera	9	12.86	—	—	History of injection treatment for Bilharziasis	Yes	16	22.86	8	5. 71
	Dark urine	9	12.86	—	—						

UD: undetermined; below detection level in patients who were under treatment for HCV; ^*∗*^more than one symptom was allowed; ^*∗∗*^this included IV, IM, and needle stick injury.

**Table 2:** Comparison between household contacts to HCV-positive cases and household contacts to HCV-negative cases.

Variable		Household contacts to HCV-positive/UD cases (number = 124)		Household contacts to HCV-negative (number = 16)		OR (95% CI)	Variable		Household contacts to HCV-positive/UD cases (number = 124)		Household contacts to HCV-negative (number = 16)		OR (95% CI)
		Number	%	Number	%				Number	%	Number	%	
Sex	Females	51	41.13	6	37.5	0.86 (0.24–2.81)	Condom use	No	122	98.39	16	100.0	—
	Males	73	58.87	10	62.50			Yes	2	1.61	0	0.0	
Age (years)	Mean ± SD (range)	32.89 ± 16.74 (3–75)		36.25 ± 17.07 (12–62)		*P*=0.45^a^	Shaving at community barber (males only)	No	51	41.13	8	50.0	1.43 (0.43–4.68)
Occupation	Housewife	36	29.03	3	18.75	1.00		Yes	73	58.87	8	50.0	
	Specialist	38	30.65	7	43.75	0.45 (0.07–2.19)	Circumcision	By medical personnel	22	17.74	5	31.25	1.00
	Medical/paramedical	4	3.23	1	6.25	0.33 (0.02–21.82)		By nonmedical personnel	79	63.71	10	62.50	1.79 (0.43–6.49)
	Student	38	30.65	5	31.25	1.4 (0.34–6.09)		No	23	18.55	1	6.25	5.23 (0.51–257.73)
	Worker	8	6.45	0	0.0	—							
Educational level	Illiterate/read and write	83	66.94	13	81.25	1.00	Nail trimming	Private tool	98	79.03	15	93.75	3.98 (0.56–173.85)
	Basic/secondary education	30	24.19	1	6.25	4.70 (0.65–206.20)							
	High education	11	8.87	2	12.50	0.86 (0.16–8.88)		Common tools	26	20.97	1	6.25	
Marital status	Unmarried	45	36.29	5	31.25	0.80 (0.20–2.69)	Smoking shisha	No	119	95.97	15	93.75	0.63 (0.06–31.77)
	Married	79	63.71	11	68.75			Yes	5	4.03	1	6.25	
Residence	Urban	103	83.06	15	93.75	3.06 (0.42–134.77)	Sharing razors	No	52	41.94	8	50.0	1.38 (0.42–4.53)
	Rural	21	16.94	1	6.25			Yes	72	58.06	8	50.0	
Relation to cases	Partners	9	7.26	0	0.0	1.00	Exposed to used syringe	No	45	36.29	1	6.25	**0.12 (0.003–0.82)** ^*∗*^
	Parents	30	24.19	7	43.75	0 (0–2.03)		Yes	79	63.71	15	93.75	
	Sons	66	53.23	6	37.50	0 (0–5.17)	Exposed to used toothbrush	No	106	85.48	16	100.0	—
	Siblings	10	8.06	3	18.75	0 (0–1.71)		Yes	18	14.52	0	0.0	
	Others	9	7.26	0	0.0	—	Practice of tattooing	No	108	87.10	14	87.50	1.04 (0.20–10.24)
Crowding index (person/room)	Mean ± SD (range)	1.15 ± 0.7 (0.28–3)		1.1 ± 0.5 (0.6–1.67)		*P*=0.78^a^		Yes	16	12.90	2	12.50	
History of blood transfusion	No	111	89.52	16	100.0	—	Exposed to blood	No	102	82.26	12	75.00	0.65 (0.17–3.02 A)
	Yes	13	10.48	0	0.0			Yes	22	17.74	4	25.00	
Prior surgical procedures	No	15	12.10	1	6.25	0.48 (0.01–3.64)	History of Bilharziasis	No	118	95.16	10	62.50	**0.08 (0.02–0.39)** ^*∗∗*^
	Yes	109	87.90	15	93.75			Yes	6	4.84	6	37.50	
Prior hospitalization	No	86	69.35	12	75.00	1.32 (0.37–5.99)	History of injection treatment for Bilharziasis	No	117	94.35	15	93.75	0.90 (0.10–43.10)
	Yes	38	30.65	4	25.00			Yes	7	5.65	1	6.25	

UD: undetermined; below detection level in patients who were under treatment for HCV; ^a^Student *t*-test; *P*: probability; OR (95% CI): odds ratio (95% confidence interval); ^*∗*^*P* < 0.05; ^*∗∗*^*P* < 0.01.

**Table 3:** Comparison between HCV-positive and HCV-negative household contacts.

Variable		HCV-positive/UD (number = 28)		HCV-negative (number = 112)		OR (95% CI)	Variable		HCV-positive/UD (number = 28)		HCV-negative (number = 112)		OR (95% CI)
		Number	%	Number	%				Number	%	Number	%	
Sex	Females	14	50.0	69	61.61	1.60 (0.64–4.01)	Condom use	No	28	100.0	110	98.21	0 (0–7.84)
	Males	14	50.0	43	38.39			Yes	0	0.0	2	1.79	
Age (years)	Mean ± SD (range)	47.96 ± 13.87 (16–75)		29.58 ± 15.36 (3–65)		*P* < 0.001^a^	Shaving at community barber (males only)	No	7	25.00	52	46.43	**2.6 (0.96–7.79)** ^*∗*^
Occupation	House wife	7	25.0	32	28.57	1.00		Yes	21	75.0	60	53.57	
	Specialist	16	57.14	29	25.89	2.52 (0.83–8.25)	Circumcision	By medical personnel	4	14.29	23	20.54	1.00
	Medical/paramedical	1	3.57	4	3.57	1.14 (0.02–14.19)		By nonmedical personnel	22	78.57	67	59.82	1.89 (0.55–8.29)
	Student	1	3.57	42	37.5	**0.11 (0.002–0.94)** ^*∗*^		No	2	7.14	22	19.64	0.52 (0.04–4.13)
	Worker	3	10.71	5	4.46	2.74 (0.34–18.08)							
Educational level	Illiterate/read and write	19	67.86	77	68.75	1.00	Nail trimming	Private tool	4	14.29	22	19.64	1.47 (0.44–6.39)
	Basic/secondary education	6	21.43	25	22.32	0.97 (0.28–2.91)		Common tools	24	85.71	90	80.36	
	High education	3	10.71	10	8.93	1.21 (0.19–5.36)							
Marital status	Unmarried	4	14.29	46	41.07	**4.18 (1.30–17.54)** ^*∗∗*^	Smoking shisha	No	27	96.43	107	95.54	0.79 (0.02–7.53)
	Married	24	85.71	66	58.93			Yes	1	3.57	5	4.46	
Residence	Urban	22	78.57	96	85.71	1.64 (0.47–5.05)	Sharing razors	No	9	32.14	51	45.54	1.76 (0.69–4.82)
	Rural	6	21.43	16	14.29			Yes	19	67.86	61	54.46	
Relation to cases	Partners	9	32.14	28	25.00	1.00	Exposed to used syringe	No	8	28.57	38	33.93	1.28 (0.48–3.69)
	Parents	6	21.43	3	2.68	**6.22 (1.02–44.40)** ^*∗*^		Yes	20	71.43	74	66.07	
	Sons	3	10.71	69	61.61	**0.13 (0.02–0.60)** ^*∗∗*^	Exposed to used toothbrush	No	26	92.86	96	85.71	0.46 (0.05–2.18)
	Siblings	9	32.14	4	3.57	**7.00 (1.45–37.43)** ^*∗∗*^		Yes	2	7.14	16	14.29	
	Others	1	3.57	8	7.14	0.39 (0.01–3.72)	Practice of tattooing	No	27	96.43	99	88.39	0.28 (0.01–2.05)
Crowding index (person/room)	Mean ± SD (range)	1.9 ± 0.6 (0.3–3)		1.67± 0.5 (0.3–3)		**P** **=0** **.0** **4** ^**a**^		Yes	1	3.57	13	11.61	
History of blood transfusion	No	21	75.0	106	94.64	**5.89 (1.50–23.18)** ^*∗∗*^	Exposed to blood	No	19	67.86	97	86.61	**3.06 (1.01–8.75)** ^*∗*^
	Yes	7	25.0	6	5.36			Yes	9	32.14	15	13.39	
Prior surgical procedures	No	3	10.71	13	11.61	1.09 (0.27–6.43)	History of Bilharziasis	No	22	78.57	106	94.64	**4.82 (1.15–19.63)** ^*∗*^
	Yes	25	89.29	99	88.39			Yes	6	21.43	6	5.36	
Prior hospitalization	No	16	57.14	82	73.21	2.05 (0.78–5.23)	History of injection treatment for Bilharziasis	No	25	89.29	107	95.54	2.57 (0.37–14.11)
	Yes	12	42.86	30	26.79			Yes	3	10.71	5	4.46	

UD: undetermined; below detection level in patients who were under treatment for HCV; ^a^Student *t*-test; *P*: probability; OR (95% CI): odds ratio (95% confidence interval); ^*∗*^*P* < 0.05; ^*∗∗*^*P* < 0.01.

**Table 4:** Logistic regression of being HCV-positive household contact conditioned on exposure to HCV-positive cases, relation to cases, age, educational level, and history of blood transfusion.

Variable	OR	95% CI	*P*
*HCV-positive cases vs. HCV-negative cases*	0.14	0.02–0.75	0.02
*Relation to case*			
Partners	1.00	—	—
Parents	11.35	1.65–77.90	0.01
Sons	1.26	0.16–10.15	0.83
Siblings	15.33	2.80–83.75	0.002
Others	1.22	0.06–23.21	0.89
*Age (years)*	1.08	1.02–1.15	0.01
*Educational level*			
Illiterate/read and write	1.00	—	—
Basic/secondary education	6.14	1.20–31.28	0.03
High education	1.52	0.20–11.39	0.68
*History of blood transfusion*			
Yes vs. no	8.08	1.75–37.30	0.007

OR: odds ratio; 95% CI: 95% confidence interval; *P*: probability.

## Data Availability

The data used to support the findings of this study are available from the corresponding author upon request.
